# Percutaneous endoscopic *versus* surgical gastrostomy in patients with benign and malignant diseases: a systematic review and meta-analysis

**DOI:** 10.6061/clinics/2016(03)09

**Published:** 2016-03

**Authors:** José Gonçalves Pereira Bravo, Edson Ide, Andre Kondo, Diogo Turiani Hourneaux de Moura, Eduardo Turiani Hourneaux de Moura, Paulo Sakai, Wanderley Marques Bernardo, Eduardo Guimarães Hourneaux de Moura

**Affiliations:** Hospital das Clínicas da Faculdade de Medicina da Universidade de São Paulo (HCFMUSP), Departamento de Gastroenterologia, Unidade de Endoscopia Gastrointestinal, São Paulo/SP, Brazil

**Keywords:** Gastrostomy, Mortality, Complication, Surgical Gastrostomy, Percutaneous Endoscopic Gastrostomy

## Abstract

To compare the complications and mortality related to gastrostomy procedures performed using surgical and percutaneous endoscopic gastrostomy techniques, this review covered seven studies. Five of these were retrospective and two were randomized prospective studies. In total, 406 patients were involved, 232 of whom had undergone percutaneous endoscopic gastrostomy and 174 of whom had undergone surgical gastrostomy. The analysis was performed using Review Manager. Risk differences were computed using a fixed-effects model and forest and funnel plots. Data on risk differences and 95% confidence intervals were obtained using the Mantel-Haenszel test. There was no difference in major complications in retrospective (95% CI (-0.11 to 0.10)) or randomized (95% CI (-0.07 to 0.05)) studies. Regarding minor complications, no difference was found in retrospective studies (95% CI (-00.17 to 0.09)), whereas a difference was observed in randomized studies (95% CI (-0.25 to -0.02)). Separate analyses of retrospective and randomized studies revealed no differences between the methods in relation to mortality and major complications. Moreover, low levels of minor complications were observed among endoscopic procedures in randomized studies, with no difference observed compared with retrospective studies.

## INTRODUCTION

The use of gastrostomy has expanded over the past decade, and new techniques have been developed that have made the procedure simpler and less risky 1. Gastrostomy is specifically a technique that allows direct access to the stomach to provide food to disabled patients for several reasons. Most commonly, this condition occurs in patients with neurological diseases, impairment following a stroke or obstructive head and neck tumors 1,2.

The absence of systematic reviews and meta-analyses directly comparing endoscopic gastrostomy and surgical gastrostomy (SG) techniques for all pathologies, whether benign or malignant, in the literature was one of the main reasons for conducting the present comparative review of surgical and endoscopic methods for all pathologies that may result in gastrostomy, taking into account major and minor complications. Two related reviews were found in the literature, but both of these considered specific pathologies. The first, carried out by Grant et al. 9, investigated gastrostomy complications in patients with head and neck tumors and compared radiological methods with endoscopy, not considering the surgical method. The other, by Burkitt et al. 2, similarly compared complications in patients with head and neck tumors but did not address other pathologies and only compared radiological techniques with endoscopy, given that endoscopic gastrostomy has become the technique of choice for carrying out the procedure, with SG only used in cases where the endoscopic procedure is not viable. Most studies associate SG with higher rates of complications and mortality 4,7.

Several techniques for gastrostomy tube insertion have been described. These techniques include percutaneous endoscopic gastrostomy (PEG) and SG (open SG (OSG) or surgical laparoscopic gastrostomy (SLG)) 2. SG was initially suggested in 1837 by Egeberg, a Norwegian surgeon and the first successful gastrostomy was carried out nearly 40 years later, in 1876, by Verneuil in Paris, France 1,2,3.

The SLG method also avoids the need for a laparotomy but still requires general anesthesia. Although not an absolute contraindication, prior upper abdominal surgery may make the SLG method difficult and risky 8. The SLG method additionally offers better exposure of the stomach than the open technique, in which the incision is usually quite small. However, PEG has nearly entirely displaced SG in clinical practice because the PEG procedure can be carried out more easily and without general anesthesia, which is beneficial for the usually elderly, high-risk patient population. In addition, PEG avoids the mortality and morbidity associated with laparotomy. Despite the minor invasiveness of endoscopic placement of percutaneous feeding tubes, complications remain an important problem 2,3.

There is widespread acceptance of PEG as the insertion technique of choice owing to its simplicity and effectiveness, but certain patients are not candidates for an endoscopic approach 10. The present review provides greater evidence in the current context as to which of these procedures is associated with major complications and mortality.

## METHODS

### Protocol and registration

The present review was conducted in accordance with the PRISMA (Preferred Reporting Items for Systematic reviews and Meta-Analyses) recommendations 7 and it has been registered in the PROSPERO international database (www.crd.york.ac.uk/prospero/) under number CRD42015016493 20.

### Eligibility criteria

1. Types of studies: randomized controlled trials and retrospective studies.
2. Type of participant: patients undergoing gastrostomy.
3. Types of interventions: PEG (intervention) and SG (comparison).
4. Types of outcome measures: the main outcome parameters were minor and major complications and mortality directly related to the procedure.

There is no literature on the exact classification of complications related to gastrostomy and authors have classified these complications in various ways. The methods used here are those found most often in published studies. Complications may be secondary to endoscopic procedures or directly related to gastrostomy, such as cardiopulmonary complications, hypoxemia, phlebitis, bacteremia, perforation and bleeding. Minor complications are treated conservatively. Major complications may require hospitalization 9,2,22, blood transfusions, or endoscopic or surgical therapy. The period in which complications occur may be early (until 15 days) or late (after 15 days) 10. For the present review, both major and minor complications were considered, whether early or late 2,12,22.

**Major complications.** Bowel perforation, Gastrointestinal hemorrhage, Gastrocutaneous fistula, Intra-abdominal abscess, Peristomal abscess, Peritonitis requiring surgery, Loss of catheter tract, Aspiration pneumonia, Sepsis, Buried bumper syndrome, Early inadvertent removal of tube.

**Minor complications.** Dislodged tubes, Inadvertent removal of tubes, Tube malfunction, Other tube problems conservatively managed, Peristomal leaks, Peristomal infection, Mild skin necrosis, Wound granulation, Minor wound bleeding, Wound hematoma, Post-procedure ileus, Symptomatic pneumoperitoneum, Subcutaneous emphysema, Regurgitation, Unsuccessful procedure.

### Information sources

The electronic databases searched were MEDLINE (via PubMed), Embase, Scopus, LILACS, the Cochrane Library (via BVS), and CINAHL (via EBSCO), from inception until February 2015.

### Search

The descriptors used for the study were as follows: ((Gastrostomies OR Gastrostomie OR Gastrostomy OR Percutaneous endoscopic gastrostomy)) AND random*.

### Study selection

The process of including or excluding studies according to PRISMA is presented as a flow chart. The eligibility assessment and selection of records shown were performed independently in a standardized manner by two reviewers. Disagreements between the reviewers were resolved by consensus and the search was conducted in all languages, with no limit regarding time.

Studies comparing patients undergoing endoscopic gastrostomy and SG were included regardless of cause and enrolled patients aged over 18 years and with a minimum period of one month of follow-up. Abstracts, letters, editorials, expert opinions, case reports and reviews were excluded. Studies that did not consider the desired outcomes or that compared other techniques were also not included.

### Data collection process and items

Data were extracted independently by two reviewers (JGPB, BWM) using forms (checklists) that are standard for cohort studies 8 and randomized clinical trials 7. The data were drawn from all studies comparing endoscopic gastrostomy and SG using the following as main variables: endoscopic gastrostomy, gastrostomy, follow-up, early and late complications and minor and major complications. Complications not related to the procedure, mortality not related to the procedure, were excluded.

### Risk of bias

Publication bias was assessed by two independent reviewers and retrospective and randomized studies were evaluated separately. For retrospective studies, the Newcastle scale 18 was used based on the Jadad score for cohort studies and randomized studies 11. The strength of evidence was evaluated using the Oxford Centre's recommendations for evidence-based medicine 19. The results of the evaluation were shown to be valid and reliable. Potential confounding factors were identified, as many authors do not take these important aspects into account in their analyses, which can lead to bias. The primary outcome measures used had also to be clearly indicated in the study. If they were not indicated or if the study based its findings on key secondary endpoints, the study was rejected.

### Planned methods of analysis

The analysis was performed using Review Manager (RevMan) 5.3 21 from the Cochrane Informatics & Knowledge Management Department website. Risk differences for dichotomous variables were computed using a fixed-effects model and the respective forest and funnel plots were obtained. Data on risk differences and the 95% confidence intervals (CIs) for each outcome were calculated using the Mantel-Haenszel test. Inconsistency (heterogeneity) was qualified and reported using the Chi-squared (Chi^2^) and Higgins methods and was termed I^2^. Data on the absolute risk reduction (ARR) or increase (ARI) and the number needed to treat (NNT) or harm (NNH) were obtained for validity and applicability using Critically Appraised Topics (CAT) software 19.

## RESULTS

**Figure 1:**
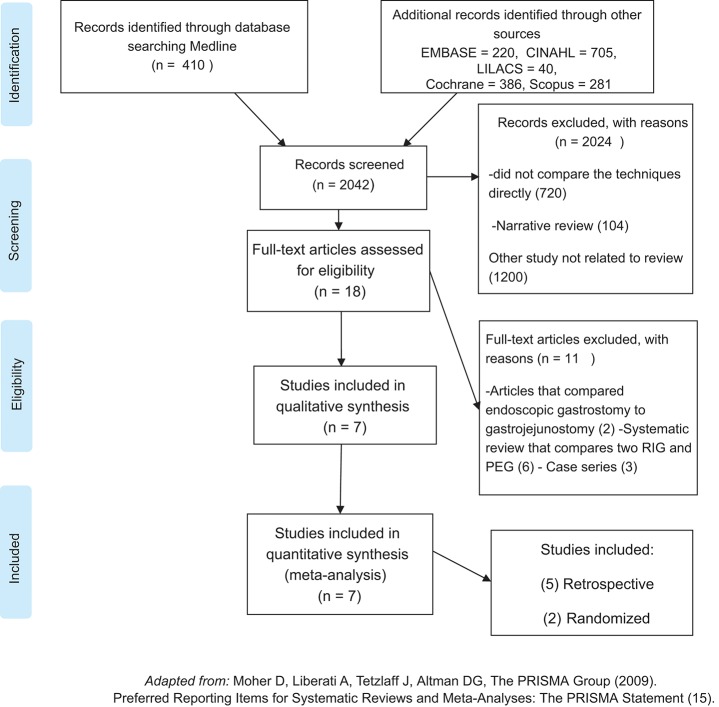
Search strategy and selection of studies.

Initially, 2,042 studies were retrieved. A total of 2,024 studies were excluded for various reasons after reading: 720 presented no direct comparison of the techniques under study, 104 were narrative reviews and another 1,200 had no direct relation to the review. For evaluation and eligibility, the full text of the remaining eighteen articles was read and eleven studies were further excluded for various reasons: two studies compared endoscopic gastrostomy with gastrojejunostomy, six were comparative reviews of radiological and endoscopic techniques and three were case series. Selected a total of seven studies of the remaining studies, two were prospective randomized studies and five were retrospective cohort studies. The retrospective and randomized studies were evaluated independently. Few randomized studies compared one technique with the other. All strategies and the selection procedure are represented in the diagram below (Figure 1).

### Study characteristics

Seven studies were included for review, including two randomized controlled trials and five retrospective cohort studies. The total population was 406 individuals, with 232 having undergone endoscopic gastrostomy and 172 having undergone SG. The main indications were neurological; traumatic; tumors of the head and neck; and other situations, such as stenosis or esophageal atresia (Table 1). All studies used the Gauderer-Ponsky or “pull” endoscopic gastrostomy technique described in 1980 17,24, 27. Certain studies did not mention whether the patients received antibiotic prophylaxis. The main outcomes studied were procedure-related complications, divided into major, minor and mortality complications directly related to the procedure.

### Risk of bias within studies

For the prospective randomized studies, the Jadad score 11, ranging from 0 to 5, was used and only the studies with a Jadad score ≥3 were selected. For observational studies, the Newcastle-Ottawa rating scale 12 was used and only studies with a score ≥6, of a maximum total of 9 points, were selected. Publication bias is related to what is likely to be published among what is available to be published (Tables 4 and 5, Supplementary file).

### Results of individual studies

Of the 406 patients in total, 232 had undergone PEG and 174 had undergone SG. Among these patients, 27 major complications were observed, 16 of which were related to the endoscopic procedure and 11 of which were related to the surgical procedure. Minor complications occurred in 57 patients with SG and in 56 with PEG. Moreover, mortality related to the procedures was higher in the group with SG (five cases) compared with the group with PEG (one case).

### All complications in retrospective studies

In the retrospective studies, with a sample of 205 individuals, complications occurred in 125 for PEG and in 77 for SG 23,4,6,16,29. There was no significant difference favoring either group.

### Major complications in retrospective studies

Of a total of 18 complications, 12 were for PEG and 6 were for SG. There was no significant difference between the four studies. Only one study 29 showed a significant difference (95% CI (0.333 to 0.547), ARR=10.7%, NNT=9, i.e., it would be necessary to treat nine patients for one to have a higher number of complications) (Table 2). Analysis of the retrospective studies 23,4,6,16,29 showed that the most frequent major complications were peritonitis requiring surgical intervention, aspiration and sepsis, with certain cases resulting in death.

### Minor complications in retrospective studies

Of 59 minor complications reported in the retrospective studies, 31 were for PEG and 28 were for SG; this difference was not significant (Table 2). The most frequent complications were small tube leaks, stoma leakage, displacement of the tube and superficial cellulitis. There was no death related to minor complications in most studies (Table 2) 23,4,6,16,29.

### Mortality related to the procedure in retrospective studies

Three deaths related to the procedure occurred, all of which were following SG. The leading causes of death were peritonitis and aspiration pneumonia. The risk difference analysis did not show a statistically significant trend favoring any group. Mortality occurred in only three retrospective studies (Table 2) 23,6,16.

### All complications in randomized studies

Among the 201 patients in total, there were 104 complications for PEG and 97 for SG. In all, 29 complications were related to PEG and 42 were related to SG. There was no significant difference between the groups (Table 3).

### Major complications in randomized studies

Four complications occurred in the PEG group and 5 occurred in the SG group. There was no significant difference between PEG and SG. The most common major complications were pneumonia and peritonitis (Table 3).

### Minor complications in randomized studies

Of 52 complications, 25 were related to PEG and 27 were related to SG. The study by Ljungdahl et al. 14 showed that PEG was associated with significantly fewer minor complications compared with SG (95% CI (0.124 to 0.562), ARR = 0.343, NNT=3, i.e., it would be necessary to treat three patients for minor complications). The most common complications were leaks around the tube, wound infection, dislodged tubes, and stoma leakage (Table 3).

### Procedure-related mortality in randomized studies

Three deaths were related to the procedures, with one in the PEG group and two related to SG in the study by Ljungdahl et al. 14. In contrast, in the study by Stiegmann et al. 26, there were no deaths related to the procedure. The main cause of death was aspiration pneumonia. However, there was no significant difference between PEG and SG (Table 3).

### Summary of results (meta-analyses) Risk of bias across studies and additional analyses

#### Risk of bias across studies and additional analyses

The data on effect estimates and CIs for each study are illustrated graphically below. The numerical group-specific summary information, effect sizes, CIs and percentage weights are also presented in the following tables. Sensitivity analysis was carried out using the heterogeneity test and is represented in the form of forest and funnel plots.

**Figure 2:**
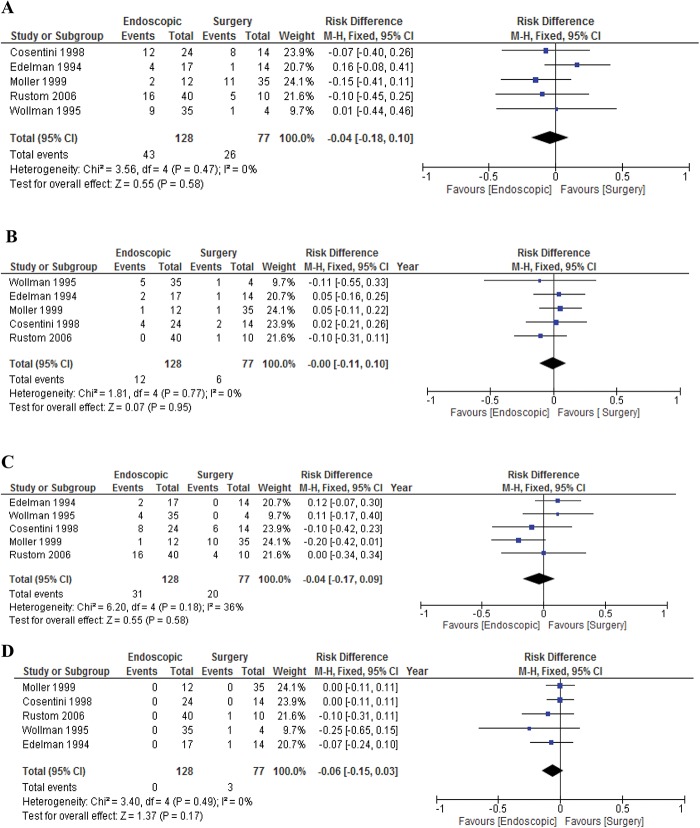
Summary of meta-analysis of retrospective studies. (A) meta-analysis--all complications in retrospective studies. (B) meta-analysis of major complications in retrospective studies. (C) meta-analysis of minor complications in retrospective studies. (D) meta-analysis of mortality related to procedures in retrospective studies.

There was no statistically significant difference between PEG and SG (risk difference = -0.04, 95% CI (-0.18 to 0.10), Figure 2a). For major and minor complications in particular, there was no difference between PEG and SG (risk difference = -0.00, 95% CI (-0.11 to 0.10)), Figure 2b and risk difference = -0.04, 95% CI (-0.17 to 0.09), Figure 2c, respectively). Additionally, for mortality related to the procedures (Figure 8), there was no difference between PEG and SG (risk difference = -0.06, 95% CI (-0.15 to 0.03), Figure 2d). Sensitivity analysis for retrospective studies (Figure 4Figure 5Figure 6Figure 7).

**Figure 3:**
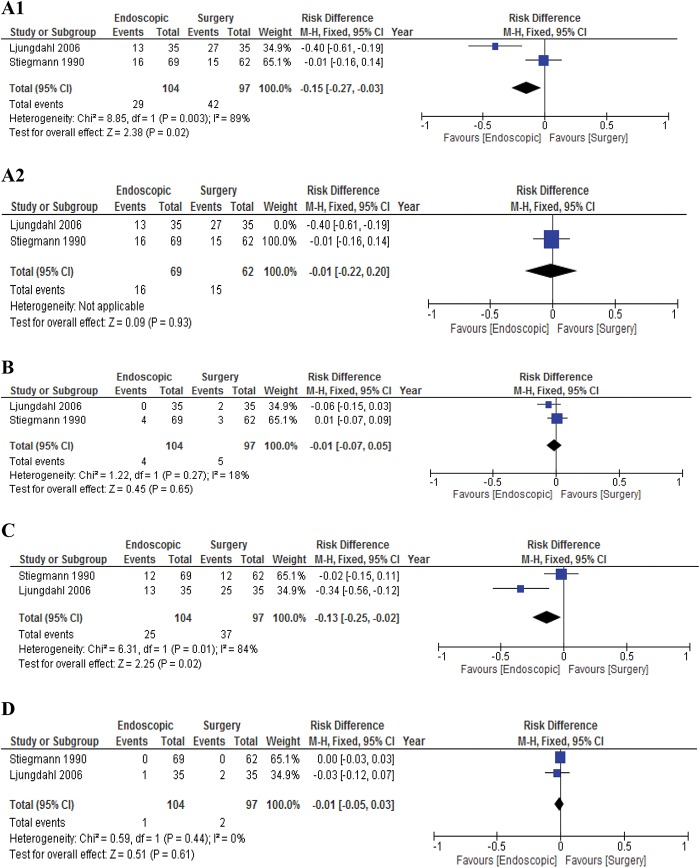
Summary of meta-analysis of randomized studies. (A1-A2) meta-analysis of all complications for randomized trials. (B) meta-analysis of major complications in randomized studies. (C) meta-analysis of minor complications in randomized studies. (D) meta-analysis of mortality related to procedures in randomized studies.

**Figure 4:**
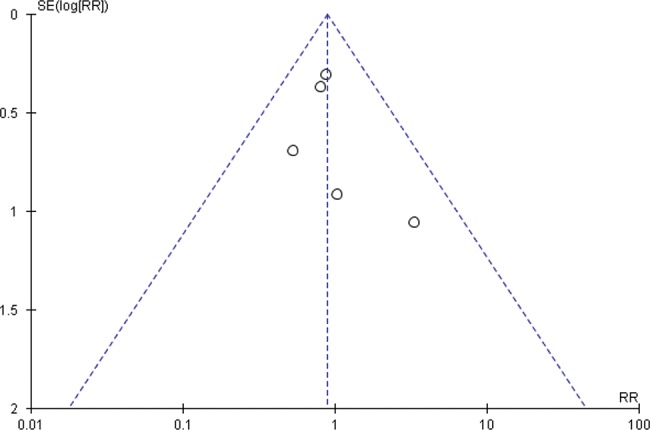
Funnel plot of all complications in retrospective studies. All studies are inside the funnel plot.

**Figure 5:**
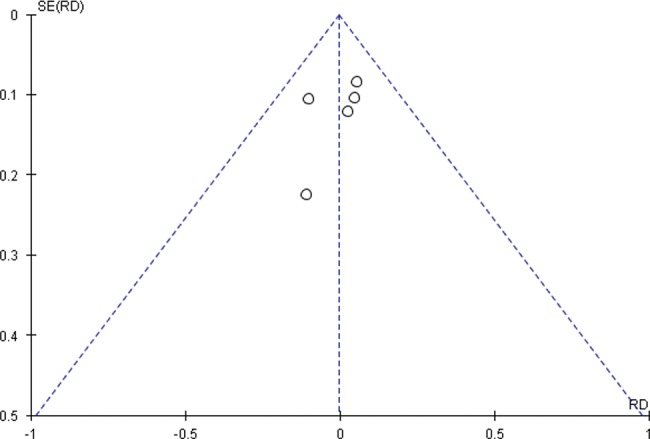
Funnel plot of major complications in retrospective studies. All studies are inside the funnel plot.

**Figure 6:**
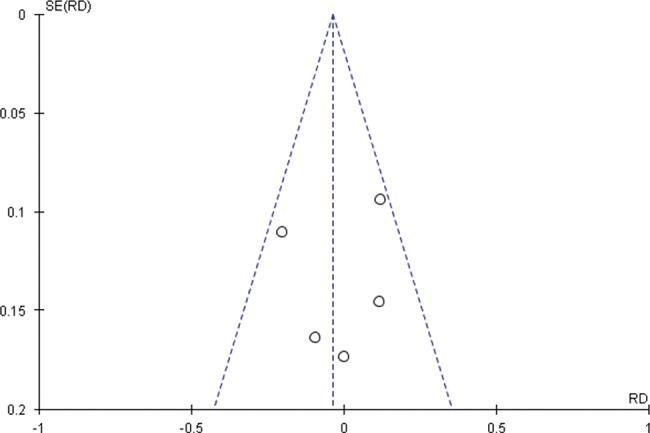
Funnel plot of major complications in retrospective studies. All studies are inside the funnel plot.

**Figure 7:**
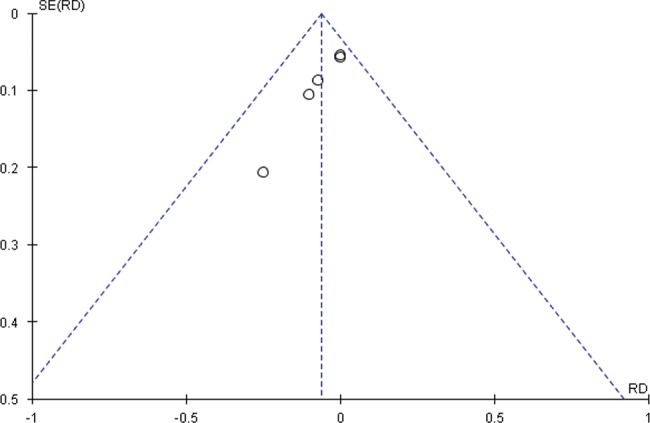
Funnel plot of mortality related to procedures in retrospective studies. All studies are inside the funnel plot.

**Figure 8:**
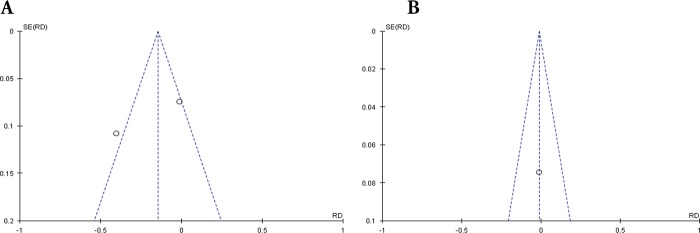
Funnel plot of all complications in randomized studies. The study by Ljungdahl 3 was outside of the funnel, which could indicate important bias in the interpretation (Figure 8a, Supplementary file) that PEG is associated with fewer complications. Furthermore, its weight in the analysis was 34% lower compared with the value in the study by Stiegmann 14, or 65.1%, which led to a new analysis of sensitivity and exclusion of the study by Ljungdahl 3 (Figure 8b, Supplementary file).

In the randomized studies related to endoscopic gastrostomy, the procedure was associated with significantly fewer complications (risk difference = -0.15, 95% CI (-0.27 to -0.03)), although high heterogeneity (I^2^=89%) was present (Figure 3a1-a2). The study by Ljungdahl 3 was outside of the funnel, which could indicate important bias in the interpretation (Figure 8a, Supplementary file) that PEG is associated with fewer complications. Furthermore, its weight in the analysis was 34% lower compared with the value in the study by Stiegmann 14 or at 65.1%, which led to a new analysis of sensitivity and exclusion of the study by Ljungdahl 3 (Figure 8b, Supplementary file). No significant difference was found (risk difference = -0.01, 95% CI (-0.22 to 0.20)) between PEG and SG (Figure 3a1-a2).

**Figure 9:**
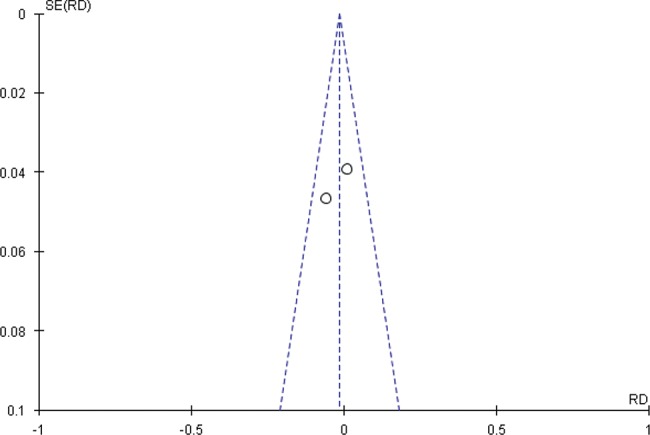
Funnel plot of major complications in randomized studies. All studies are inside the funnel plot.

**Figure 10:**
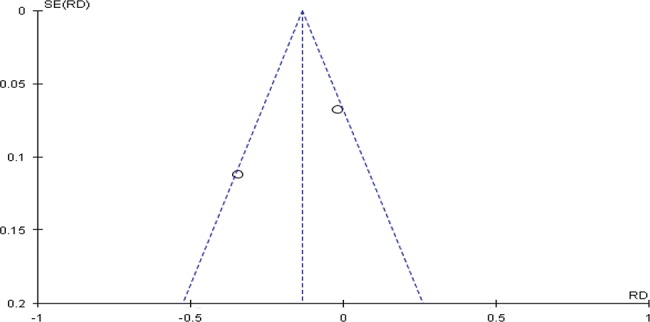
Funnel plot of minor complications in randomized studies. The heterogeneity between studies was high (Figure 11, Chi^2^=6.31, DF=1 (*p*=0.01), I^2^=84%), which may have been due to the varying characteristics of the studies, times of publication and distributions of the populations as well as other population differences. However, two studies were found to lie inside the funnel plot, demonstrating true heterogeneity.

Regarding major complications in the randomized studies, the forest plot demonstrated no significant difference between PEG and SG (risk difference = -0.01, 95% CI (-0.07 to 0.05), Figure 3b). In sensitivity analysis funnel plot of major complications in randomized studies. All studies are inside the funnel plot (Figure 9). Regarding minor complications in the randomized studies, the forest plot showed that PEG was associated with significantly fewer complications compared with SG (risk difference = -0.13, 95% CI (-0.25 to -0.02)). There was great heterogeneity between studies, which may have been due to the varying characteristics of the studies, times of publication and distributions of the populations as well as other population differences (Figure 3c). In the analysis of sensitivity, two studies were found to lie inside the funnel plot, demonstrating true heterogeneity (Figure 10, Supplementary file). Regarding mortality related to the procedures in the randomized studies, there was no significant difference between PEG and SG (risk difference = -0.01, 95% CI (-0.05 to 0.03), Figure 3d).

### DISCUSSION

The aim of the present systematic review and meta-analysis was to compare the complications and mortality directly related to PEG and SG. Seven studies were included, namely, two prospective randomized studies and five retrospective studies and these were evaluated differently, given that there are few published randomized studies comparing the two techniques. The review demonstrates, via separate analyses of the randomized trials, that endoscopic gastrostomy has a low rate of minor complications compared with SG. In contrast, the retrospective studies exhibited no significant differences.

Endoscopic gastrostomy is used as the method of choice in nearly all centers worldwide, replacing SG 24,25,30. Many studies indicate that SG is associated with more complications and higher mortality, mainly because it is a more invasive procedure with a longer recovery period. In addition, this method is more expensive and involves operating room reservations and an anesthesia team in 100% of cases and in certain cases, patients need intensive care. Although more practical, SG is also associated with complications and mortality. In particular, several studies have reported many complications and considerable cases of mortality linked to this procedure and there have additionally been many unreported cases of complications and mortality directly associated with the procedure 12,13. Many of these complications and cases of mortality involve seriously ill patients, the bedridden, or the elderly, with multiple comorbidities during hospitalization.

Despite high heterogeneity in the randomized studies 14,26, in the sensitivity analysis, it was found that there was true heterogeneity and inconsistency (outliers outside of the funnel). There were no significant differences between the two techniques regarding major complications or mortality related to the procedure in either the retrospective or randomized studies. Analysis of overall complications in the randomized trials revealed that SG had a higher rate of complications, but when sensitivity was analyzed and discrepancies (outliers) were removed, there was no difference between the two techniques.

Another problem is the lack of adequate standardization in the literature regarding the definitions of major and minor complications, which has also been a key factor in increasing bias. Both SG and endoscopic gastrostomy are associated with many complications, large and small. Specifically, there are many reports of complications that led to the death of patients as a result of associated diseases. For example, Grant et al. 9 assessed complications of PEG in patients with head and neck tumors; a total of 253 gastrostomy cases were observed, with 1% of deaths, 3.3% of minor complications and 28.9% of major complications related to PEG. Additionally, many studies do not report whether they used antibiotics for prophylaxis, which reduces complications and mortality from infections. In a study by Lipp et al. 1, 1,100 patients in ten randomized clinical trials were evaluated and the use of antibiotic prophylaxis was found to reduce complications related to infections 1. In the present review and meta-analysis, the main major complications reported were pneumonia aspiration, sepsis, and peritonitis, and the main minor complications were wound infection, probe displacement, and leakage at the site of the puncture.

#### Limitations

- Few randomized studies are available in the literature
- There is a lack of recent studies comparing the two techniques using the means available today
- There is a lack of uniformity in surgical techniques in studies
- Certain studies do not mention whether they used antibiotic prophylaxis or not
- Many retrospective studies have a small population
- There is a lack of standardization regarding major and minor complications

This review indicates that PEG and SG are equivalent methods based on the evidence and that, furthermore, PEG is associated with fewer comorbidities.

Separate analysis of retrospective and randomized studies revealed no differences between the methods in relation to mortality and major complications, with low levels of minor complications for endoscopic procedures in randomized studies and no difference observed when compared with retrospective studies.

## ACKNOWLEDGMENTS

This study was supported entirely by the Gastrointestinal Endoscopy Unit, Gastroenterology Department, University of São Paulo Medical School (Center of Excellence 2015-2020 World Endoscopy Organization).

## Figures and Tables

**Figure 11:**
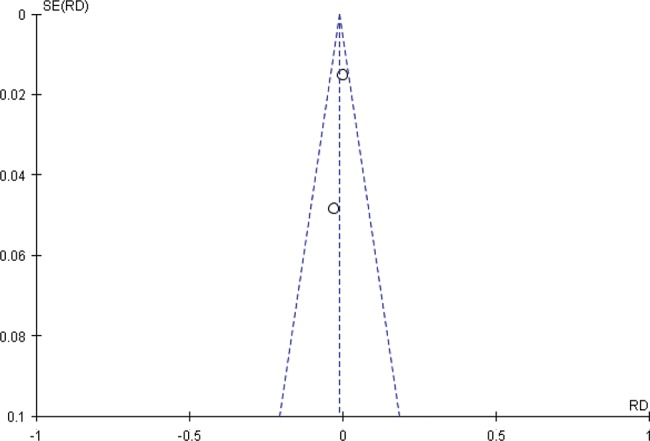
Funnel plot of mortality related to procedures in randomized studies. All studies are inside the funnel plot.

**Table 1:** Characteristics of retrospective and randomized studies.

Authors	Country	Population	Comparison	Sample (n=523)	Method	Mean age (years)	Follow-up
**Rustom et al. 23 2006 retrospective**	England	Head and neck tumors	PEG	40	Pull*	63	18 M
						SG		10	OSG*	65
**Cosentini et al. 4 1998 retrospective**	Austria	Oropharyngeal cancer, head and neck cancer, neurological disorder	PEG	35	Pull	58	17 M
						SG		4	OSG	28
**Wollman et al. 30 1995 retrospective**	USA	Neurological impairment, head and neck cancer, gastrointestinal decompression	PEG	69	Pull	61	1 M
						SG		62	OSG	54
**Muller et al. 16 1999 retrospective**	Sweden	Neurological disorder, malignant cancer, other	PEG	24	Pull	55	1 M
						SG		14	OSG	39
**Edelman et al. 6 1994 retrospective**	USA	Inability to eat, malnutrition, recurrent aspiration, head and neck or esophageal tumor, esophageal obstruction from radiation	PEG	17	Pull	81	1 M
						SG		14	SLG*	61
**Ljungdahl et al. 14 2006 randomized**	Sweden	Stroke, neurological disease, oropharyngeal cancer, cerebral trauma	PEG	35	Pull	69	45 M
						SG		35	OSG	65
**Stiegmann et al. 26 1990 randomized**	USA	Oropharyngeal cancer, head and neck cancer, esophageal stricture	PEG	12	Pull	48	4 M
						SG		35	OSG	71

OSG: open surgical gastrostomy; SG: surgical gastrostomy; PEG: percutaneous endoscopic gastrostomy; Pull: method of percutaneous gastrostomy; SLG: surgical laparoscopic gastrostomy.

**Table 2:** Statistical summary of complications and mortality for retrospective studies.

	EVIDENCE SUMMARY				
Authors	PEG INTERV/N	SG INTERV/N	ARR/ARI	95% CI	NNT/NNH
**All complications**					
*Rustom et al. 2006*	12/40	8/10	0.100(R)	-0.256 to 0.398	NS
*Cosentini et al. 1998*	16/24	5/14	0.071(R)	-0.245 to 0.445	NS
*Wollman et al. 1995*	9/35	1/41	-0.007(I)	-0.455 to 0.441	NS
*Moller et al. 1999*	2/12	1/35	-0.147(I)	-0.114 to 0.408	NS
*Edelman et al. 1994*	4/17	1/14	0.227(R)	-0.78 to 0.46	NS
	128	77			
**Major complications**					
*Rustom et al. 2006*	0/40	1/10	0.100(R)	-0.086 to 0.134	NS
*Cosentini et al. 1998*	4/24	2/14	-0.024(I)	-0.260 to 0.212	NS
*Wollman et al. 1995*	5/35	1/4	0.107(R)	0.333 to 0.547	9
*Moller et al. 1999*	1/12	1/35	-0.054(I)	-0.220 to 0.112	NS
*Edelman et al. 1994*	2/17	1/14	-0.047(I)	-0.251 to 0.157	NS
	128	77			
**Minor complications**					
Rustom et al. 2006	16/40	4/10	0.000(R)	-0.339 to 0.339	NS
Cosentini et al. 1998	8/24	6/14	0.096(R)	-0.225 to 0.417	NS
Wollman et al. 1995	4/35	0/4	0.114(R)	-0.219 to 0.009	NS
Moller et al. 1999	1/12	10/35	0.203(R)	-0.113 to 0.410	NS
Edelman et al. 1994	2/17	0/14	0.164(R)	-0.406 to 0.078	NS
	128	77			
**Procedure-related mortality**					
*Rustom et al. 2006*	0/40	1/10	0.100(R)	-0.086 to 0.286	NS
*Cosentini et al. 1998*	0/24	0/14	0(R)	0	NS
*Wollman et al. 1995*	0/35	0/4	0(R)	0	NS
*Moller et al. 1999*	0/12	1/35	0.029(R)	-0.027 to 0.085	NS
*Edelman et al. 1994*	0/17	1/14	0.071(R)	-0.064 to 0.206	NS
	128	77			

PEG: percutaneous endoscopic gastrostomy; SG: surgical gastrostomy; ARR/ARI: absolute risk reduction or increase – (R): reduction, (I): increase; 95% CI: 95% confidence interval; NNT/NNH: number needed to treat or harm, (-): negative, NS: not statistically significant; Interv: intervention.

**Table 3:** Statistical summary of complications and mortality for randomized studies.

	EVIDENCE SUMMARY				
Authors	PEG INTERV/N	SG INTERV/N	ARR/ARI	95% CI	NNT/NNH
**All complications**					
Ljungdahl et al. 2006	13/35	27/35	0.400(R)	-0.188 to 0.612	NS
Stiegmann et al. 1990	16/69	15/62	-0.010(I)	-0.136 to 0.156	NS
	104	97			
**Major complications**					
Ljungdahl et al. 2006	0/35	2/35	-0.057(I)	-0.020 to 0.134	NS
Stiegmann et al. 1990	4/69	3/62	-0.054(I)	-0.087 to 0.067	NS
	104	97			
**Minor complications**					
Ljungdahl et al. 2006	13/35	25/35	0.343(R)	0.124 to 0.562	3
Stiegmann et al. 1990	12/69	12/62	0.020(R)	-0.133 to 0.153	NS
	104	97			
**Procedure-related mortality**					
Ljungdahl et al. 2006	1/35	2/35	0. 028(R)	-0.067 to 0.123	NS
Stiegmann et al. 1990	0/69	0/62	0(R)	0	NS
	104	97			

PEG: percutaneous endoscopic gastrostomy; SG: surgical gastrostomy; ARR/ARI: absolute risk reduction or increase – (R): reduction, (I): increase; 95% CI: 95% confidence interval; NNT/NNH: number needed to treat or harm, (-): negative, NS: not statistically significant ; Interv: intervention.
